# Development and validation of a real-time PCR assay for the detection of SEA4 clade rabies virus (Philippine strain) in post-mortem non-brain animal samples

**DOI:** 10.1371/journal.pone.0362890

**Published:** 2026-11-13

**Authors:** Daria L. Manalo, Jude Karlo G. Bolivar, Jeromir G. Bondoc, Blanca J. Nagataki, Karl Ian T. Ermino, Kazunori Kimitsuki, Leilanie B. Nacion, Mark Joseph M. Espino, Chun-Ho Park, Satoshi Inoue

**Affiliations:** 1 National Reference Laboratory for Rabies and other Lyssaviruses, Veterinary Research Department, Research Institute for Tropical Medicine, Muntinlupa City, Philippines; 2 Immunology Department, Research Institute for Tropical Medicine, Muntinlupa City, Philippines; 3 Department of Microbiology, Faculty of Medicine, Oita University, Oita, Japan; 4 Department of Veterinary Pathology, School of Veterinary Medicine, Kitasato University, Towada, Aomori, Japan; 5 Department of Veterinary Science, National Institute of Infectious Diseases, Shinjuku-ku, Tokyo, Japan; 6 Center for Infectious Disease Epidemiology and Prevention Research, Tokyo University of Agriculture and Technology (Cooperative Division of Veterinary Sciences), Tokyo, Japan; Bai Jerbai Wadia Hospital for Children, INDIA

## Abstract

Rabies remains a major public health concern in endemic settings, where effective control depends on timely and reliable post-mortem confirmation of infection in animal reservoirs. Although the direct fluorescent antibody test (dFAT) remains the reference standard, its dependence on fresh brain tissue and susceptibility to specimen degradation limits its operational use in decentralized surveillance systems. The present findings demonstrate that the locally optimized RT-qPCR assay targeting the nucleoprotein gene in non-brain tissues exhibits high analytical sensitivity and strong concordance with dFAT results obtained from post-mortem brain specimens of dogs. The consistent performance in nasal planum and follicle-sinus complex samples supports using safer, more accessible post-mortem specimens, reinforcing the potential for scalable, field-adapted molecular diagnostics to strengthen routine animal rabies surveillance in endemic regions.

## Introduction

Rabies is a zoonotic disease that affects the central nervous system (CNS) and is almost invariably fatal once clinical signs appear; however, it is entirely preventable through prompt wound management and timely administration of rabies vaccine and rabies immunoglobulin as part of post-exposure prophylaxis (PEP) [1–3]. Infected dogs account for up to 99% of human rabies cases worldwide, with transmission occurring primarily through bites, although non-bite exposures—such as scratches, abrasions, or contact of open wounds with saliva or other potentially infectious material—also pose significant risk [4–6].

Despite the availability of effective vaccines, rabies remains a neglected and underreported disease, continuing to impose a major public health and economic burden in many developing countries. Globally, rabies causes an estimated 59,000 human deaths each year and results in an annual economic burden of approximately USD 8.6 billion [7]. The Philippines, one of the rabies-endemic countries in Southeast Asia, reported approximately 200–300 human rabies cases annually in 2020 [8]. Although the enactment of Republic Act No. 9482 (the Anti-Rabies Act of 2007) aimed to reduce and ultimately eliminate human rabies, the country continues to bear one of the highest rabies burdens in Asia [9]. Dogs remain the principal reservoir of infection, and dog vaccination coverage (53.3% in 2018) remains substantially below the recommended 80% threshold required to interrupt transmission [8]. Consequently, reported human rabies cases increased to more than 350 annually in 2022 and 2023, underscoring the persistent public health challenge posed by dog-mediated rabies in the Philippines [10].

Given that human rabies remains almost entirely preventable yet continues to be driven by canine transmission, effective animal rabies surveillance depends on accurate post-mortem diagnosis, which remains centered on the direct fluorescent antibody test (dFAT) as the gold standard for case confirmation, which relies on brain tissue for the detection of viral antigen [11]. Although dFAT provides rapid and reliable results under optimal conditions, its sensitivity declines markedly as brain tissue undergoes post-mortem decomposition, posing risks of false negative results, a limitation that is particularly pronounced in tropical regions where high ambient temperatures and delays in sample transport frequently compromise specimen integrity [3,12]. In tropical settings such as the Philippines, where ambient temperatures commonly reach 29–35 °C, rapid post-mortem autolysis further constrains the reliability of antigen-based diagnostics; while rabies antigen may remain detectable for several days in decomposed carcasses, diagnostic sensitivity declines substantially with progressive tissue degradation, compounded by logistical challenges in specimen collection, preservation, and transport [11,12].

In this context, follicle-sinus complexes (FSCs) of the muzzle skin and the nasal planum (NP) emerge as robust alternative post-mortem diagnostic specimens that are practical, safer for personnel, and easier to collect than brain tissue, as they eliminate the need for cranial opening during specimen collection. While brain-based assays are highly dependent on specimen integrity and proper storage, FSCs consistently retain rabies virus antigen, genome, and intact viral particles within Merkel cells in post-mortem field samples, indicating greater resilience to post-mortem degradation and handling-related compromise [13–15]. Their diagnostic suitability is further supported by centrifugal viral spread via trigeminal nerve branches to peripheral tissues, including the muzzle skin and nasal planum, particularly during late-stage infection [16].

Molecular diagnostic approaches have become increasingly central to rabies surveillance because of their superior sensitivity, scalability, and capacity for viral characterization. Rabies viral RNA demonstrates greater thermal stability than antigen, remaining detectable in naturally infected brain samples for prolonged periods under elevated temperatures, supporting the robustness of RNA-based detection in field settings [17]. However, conventional gel-based reverse transcription-PCR (RT-PCR) is prone to contamination and non-specific amplification, often necessitating sequencing for confirmation. In contrast, RT-qPCR offers enhanced sensitivity and specificity and has emerged as a cornerstone of rabies laboratory diagnosis [18]. While pan-lyssavirus assays such as LN34 perform well across diverse lyssaviruses, genetic heterogeneity among rabies virus lineages can affect assay performance when primers and probes are not optimized for locally circulating strains (SEA4 clade). Given that Philippine rabies viruses form distinct phylogenetic clusters within Asian dog-mediated lineages, regionally tailored RT-qPCR assays are critical for maximizing diagnostic sensitivity and improving surveillance accuracy in endemic settings [19–22].

In 2006, two human rabies cases were reported in Japan following dog-bite exposure in the Philippines, with clinical disease developing 2–3 months post-exposure. Molecular characterization of the causative virus involved amplification and sequencing of the rabies virus nucleoprotein (N) gene using a primer cocktail, enabling the identification of a Philippine rabies virus isolate [21]. The resulting sequence data formed the basis for the design of strain-specific primers and probes targeting this isolate.

Building on these Philippine strain–derived primers and probe, the present study further developed and optimized an RT-qPCR assay for post-mortem detection of rabies viral RNA in dFAT-tested brain samples. The assay was subsequently validated using non-brain specimens, specifically follicle-sinus complexes and nasal planum tissues. This locally optimized RT-qPCR assay offers a promising tool for detecting rabies virus strains circulating in the Philippines and is particularly suited for post-mortem diagnostic and surveillance applications when brain tissue is degraded, insufficient, or unavailable for direct fluorescent antibody testing. By enabling reliable detection from alternative specimen types, the assay has the potential to strengthen rabies diagnosis and surveillance capacity in endemic settings.

## Materials and methods

### Ethical and biosafety considerations

A biosafety clearance was obtained from the Biorisk Management Office (BRMO) of the Research Institute for Tropical Medicine (RITM, BRMO Clearance No. 221001), allowing the utilization of the study protocol. An Institutional Animal Care and Use Committee (IACUC) certification was deemed unnecessary because no live animals were used in the conduct of this study. Under Philippine regulations, IACUC certification is required only for studies involving live animals [23].

### Post-mortem sample collection

Dog heads were collected post-mortem for routine rabies diagnostic testing and submitted to the Research Institute for Tropical Medicine (Muntinlupa City, Philippines) and Regional Animal Disease Diagnostic Laboratories (RADDL) across various regions of the Philippines. Brain tissues were obtained following removal of the skull cap and incision of the dura mater, with sampling of the hippocampus, cerebellum, and brainstem. Follicle sinus complexes were collected by circumferential incision around the vibrissal hair bulb, followed by transverse sectioning at the level of the ring sinus. Nasal planum samples were obtained by midline incision of the rostral nose and transverse sectioning at the level of the alar fold, with excess tissue trimmed to yield approximately 5-mm-thick specimens [14,16]. All samples were handled under standard biosafety protocols and stored at −80 °C until RNA extraction. The post-mortem interval was not standardized, as samples originated either from routine diagnostic surveillance (RITM) or from archived materials, including frozen canine heads (RADDLs), resulting in variable and indeterminate collection intervals.

### RNA extraction

Thirty milligrams of tissue were placed in 500 µL lysis buffer in a bead-beating tube (BMS 2641-0B) containing a single 5-mm stainless steel bead (BMS SS50−0001). Samples were homogenized using a Bertin Precellys tissue homogenizer (Bertin Technologies, France) for four cycles at 8,000 rpm for 30 s, with cooling on a chilled rack (0 to −20 °C) for 50 s between cycles. Total RNA was extracted using the RNeasy Mini Kit (Qiagen, Germany) according to the manufacturer’s instructions, eluted in 50 µL RNase-free water, and stored at −80 °C until use.

### PCR controls

Rabies viral RNA was extracted from dFAT-positive brain tissues and amplified by RT-qPCR targeting the rabies virus nucleoprotein (N) gene. The resulting amplicons were pooled and purified using the QIAquick PCR Purification Kit (Qiagen, Germany). Purified rabies N-gene amplicons were quantified using a DS-11 Series UV–Vis spectrophotometer (DeNovix Inc., Wilmington, DE, USA), and the measured concentration was used to determine the DNA copy number (Thermo Fisher Scientific, n.d.). Dilutions of this stock in nuclease-free water were prepared accordingly. Varying concentrations were utilized throughout the optimization and validation process. Nuclease-free water was used in the no-template control reactions. A negative extraction control was used in the validation part of the study to rule out contamination which may result to false positives. An internal control was not incorporated into the assay in this study; therefore, sample-specific extraction efficiency and amplification inhibition were not independently monitored.

### Primers and probe

The RT-qPCR assay targeted the rabies virus nucleoprotein (N) gene using primers and probes designed from sequences of Philippine strain–derived rabies virus identified in autopsy cases in Japan [21] (Table 1).

**Table 1:** Primers and probe sequences.

Name		Sequence
Primers	RaVPh Reverse	5′-RCCTCCCAGAACAGA CATCTCA-3′
	RaVPh Forward	5’- ATGGGTCAAGTCAGRTCTCTTAATGC- 3’
Probe	RaVPh Probe	FAM- CGGTTATTGCYGCATGTGCC CCCC- TAMRA

### Optimization of qPCR conditions

Multiple parameters were evaluated to optimize the RT-qPCR assay, including primer and probe concentrations, annealing temperature, primer stability, assay specificity, limit of detection, and repeatability. A one-step probe-based real-time PCR assay was initially performed using the manufacturer-recommended reaction volumes (Table 2) and cycling conditions from the iTaq Universal Probes One-Step Kit (Bio-Rad Laboratories, USA) on a CFX96 Touch Real-Time PCR system (Bio-Rad Laboratories, USA). All parameters were assessed using positive and no-template controls performed in triplicate.

**Table 2:** RT-qPCR master mix preparation.

PCR Component	Volume (1X) (µL)
iTaq universal probe reaction mix (2X)	10.0
Forward primer (10µM)	1.0
Reverse primer (10µM)	1.0
Probe (5µM)	0.50
iScript™ reverse transcriptase	0.50
Nuclease-free water	4.0
Nucleic Acid Template	3.0 (added individually)
TOTAL	20.0

### Annealing temperature

The optimal annealing temperature was determined using a temperature gradient ranging from 56 to 65 °C, with gradient points automatically set by the CFX PCR system (56.8, 57.3, 58.4, 60.0, 61.9, 63.5, 64.4, and 64.8 °C). The optimal temperature was defined as the condition yielding 100% detection, with the lowest mean Ct value and minimal standard deviation across triplicate reactions, and no evidence of non-specific amplification [24].

### Primers and probe concentrations

The optimized temperature and the same criteria [24] were applied in determining optimal primer and probe concentrations. A primer concentration gradient (2.0, 4.0, 6.0, 8.0, and 10.0 µM) was prepared from 100 µM stock solutions. To optimize probe concentration, a gradient of 0.50, 1.0, 2.0, and 3.0 µM was employed. Primer and probe stability were subsequently assessed at the optimized annealing temperature by amplifying one positive control alongside 20 no-template controls.

The optimized thermocycling conditions (Table 3) were used in the subsequent laboratory performance validation of the assay.

**Table 3:** RT-qPCR profile for rabies N gene detection.

qPCR step	Temperature (ºC)	Time	Number of cycles
Reverse transcription	50	10 minutes	1
Enzyme activation	95	3 minutes	
Denaturation	95	30 seconds	35
Annealing and extension	60	25 seconds	

### Assay efficiency

Assay efficiency was determined by constructing a standard curve using serial dilutions of the positive control (10^6^, 10^5^, 10^4^, 10^3^, 10^2^, 10^1^, and 10^0^ copies), with one reaction prepared per dilution and amplified under optimized thermocycling conditions. Amplification efficiency (E) was calculated from the slope of the standard curve using the formula *E = (10^(−1/slope) − 1) × 100*, with acceptable efficiency defined as 90–110% and a coefficient of determination (R^2^) ≥ 0.98 [25]. For this study, assay efficiency is automatically determined by the qPCR machine.

### Limit of detection (LoD)

The sensitivity of the assay was assessed by determining its limit of detection (LoD) [25]. Serial dilutions of the positive control, ranging from 10⁶ to 10 ⁻^3^, were initially tested in triplicate per concentration. The two lowest concentrations demonstrating consistent 3/3 amplification were subsequently evaluated using 20 replicates each. The LoD was defined as the lowest concentration at which at least 19 of 20 replicates (95%) yielded detectable amplification [26].

### Analytical specificity

Analytical specificity was assessed by testing positive control alongside canine parvovirus and canine distemper virus using the optimized assay conditions. All reactions were performed in triplicate.

### Assay reproducibility

Assay reproducibility was evaluated within one week of optimization by assessing intra- and inter-laboratory performance. The optimized protocol was independently performed by different operators and on three real-time PCR instruments across three RITM reference laboratories, with reproducibility assessed across three repeated runs.

### Assay validation and verification

The developed RT-qPCR assay was validated using dFAT-confirmed samples, including 20 dFAT-positive brain, (paired with 20 FSC and 20 NP), and 30 dFAT-negative brain specimens. FSC and NP are not collected from dFAT negative specimens.

### Statistical analysis

Differences in Ct values among paired brain, follicle sinus complex, and nasal planum specimens were evaluated using the Friedman test, a non-parametric test for related samples. Statistical significance was assessed at p < 0.05. Assay performance was further evaluated by determining Positive Percent Agreement (PPA) and Negative Percent Agreement (NPA) using follicle sinus complex (FSC) and nasal planum (NP) samples corresponding to the dFAT-confirmed positive and negative brain tissues. PPA was calculated as *[true positives ÷ (true positives + false negatives)] × 100*, and NPA as *[true negatives ÷ (true negatives + false positives)] × 100*. Exact 95% confidence intervals for PPA and NPA were calculated using the binomial (Clopper–Pearson) method [25].

Additionally, to evaluate the quantitative relationship between paired specimen types, Spearman's rank correlation analysis was performed to assess the correlation between RT-qPCR cycle threshold (Ct) values obtained from brain tissue and those from follicle sinus complex and nasal planum specimens. Correlation coefficients (ρ) and corresponding p-values were calculated using paired Ct values, with statistical significance defined as p < 0.05.

## Results

An RT-qPCR assay targeting the rabies virus nucleoprotein (N) gene was developed and optimized. Based on the predefined criteria of 100% detection, lowest mean Ct value with minimal standard deviation across triplicates (R1 to R3) and absence of non-specific amplification, an annealing temperature of 60 °C was identified as optimal (Table 4). Annealing temperatures below 60 °C resulted in non-amplification of some replicates (58.4°C, 57.3°C, 56.8°C); with late, non-specific amplification in the no-template control (57.3°C, 56.8°C).

**Table 4:** Determination of optimal annealing temperature using a temperature gradient.

Annealing temperature (°C)	Ct value				% Detection	Mean Ct Value	SD
	R1	R2	R3	NTC			
64.8	18.36	18.60	18.84	NA	100	18.60	0.24
64.4	18.19	18.41	18.63	NA	100	18.41	0.22
63.5	18.18	18.38	18.58	NA	100	18.38	0.20
61.9	18.09	18.27	18.45	NA	100	18.27	0.18
**60.0**	**17.85**	**17.97**	**18.09**	NA	100	**17.97**	**0.12**
58.4	17.81	18.11	NA	NA	66.67	17.96	0.21
57.3	17.92	NA	NA	34.41	33.33	17.92	NA
56.8	17.96	NA	NA	34.69	33.33	17.96	NA

Using the optimized annealing temperature, a concentration gradient and the same criteria, the optimal primer and probe concentrations were determined to be 6.0 µM and 3.0 µM, respectively (Tables 5 and 6).

**Table 5:** Determination of optimal primer concentration using a concentration gradient.

Primer concentration(µM)	Ct value				% Detection	Mean Ct Value	SD
	R1	R2	R3	NTC			
2.0	17.07	16.25	17.07	NA	100	16.80	0.47
4.0	16.03	16.27	16.28	NA	100	16.91	0.14
6.0	16.04	15.98	16.08	NA	100	16.33	0.05
8.0	16.92	16.96	17.03	NA	100	16.97	0.06
10.0	17.04	17.11	16.81	NA	100	16.99	0.15

**Table 6:** Ct values of Rabies N gene positive control in varying probe concentrations.

Probe concentration(µM)	Ct value				% Detection	Mean Ct Value	SD
	R1	R2	R3	NTC			
0.5	20.02	NA	NA	NA	33.33	20.02	NA
1.0	18.98	NA	NA	NA	33.33	18.98	NA
2.0	18.03	18.12	18.21	NA	100	18.12	0.09
3.0	17.55	17.73	17.61	NA	100	17.63	0.08

Primer and probe stability under scaled-up preparation conditions was evaluated by testing one positive control alongside 20 no-template controls. Amplification was observed only in the positive control (Ct = 17.63), with no amplification in no-template controls, indicating maintained assay stability (Fig 1).

**Fig 1:**
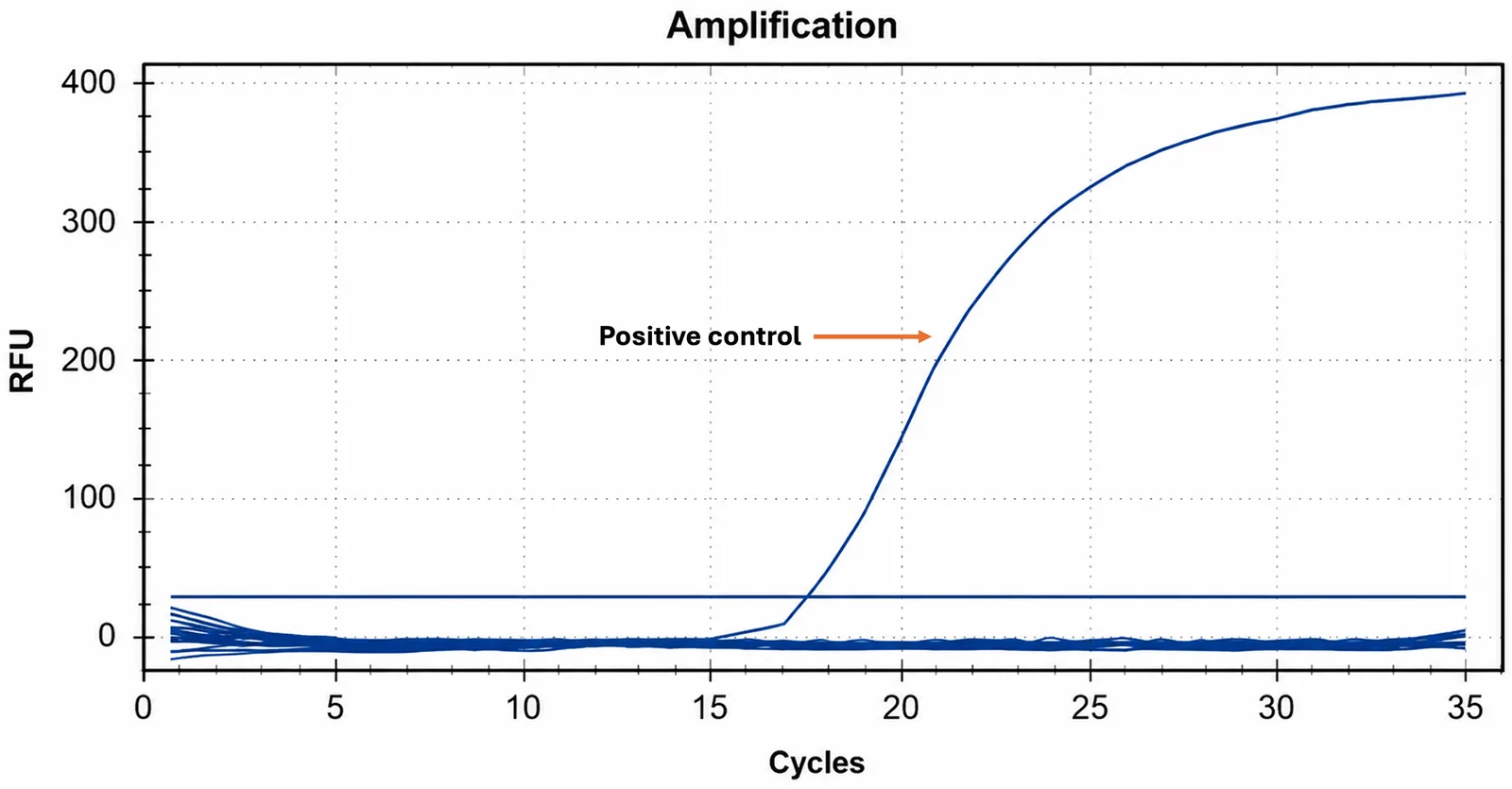
Primer and probe stability in 20-replicate NTC.

Consistent with the analytical specificity assessment, the assay detected only rabies-positive control samples, with no amplification observed in non-rabies controls (canine distemper virus and canine parvovirus) and in no-template controls, indicating absence of cross-reactivity (Table 7).

**Table 7:** Clinical specificity of the RT-qPCR assay.

Viral sample	Ct value			% Detection
	Replicate 1	Replicate 2	Replicate 3	
Rabies virus	20.21	20.19	20.35	100
Canine distemper virus	NA	NA	NA	0
Canine parvovirus	NA	NA	NA	0
No template control	NA	NA	NA	0

Assay sensitivity was evaluated using 10-fold serial dilutions of rabies viral RNA to generate a standard curve. The assay demonstrated an amplification efficiency of 106.8%, with a standard curve slope of −3.168 and a coefficient of determination (R^2^) of 0.999 (Fig 2), meeting the predefined acceptance criteria. The limit of detection was defined as the lowest dilution yielding amplification in at least 19/20 (95%) replicates and was determined to be 1 RNA copy/µL with 100% detection rate (Table 8).

**Table 8:** a. Determination of the limit of detection in different dilutions of rabies N gene positive control. b. 20-replicate testing for the determination of limit of detection.

Concentration (copies/ mL)	Ct value					% Detection
	R1	R2	R3	Mean	SD	
10^6^	12.36	12.11	12.22	12.23	0.13	100
10^5^	15.53	15.27	15.41	15.40	0.13	100
10^4^	18.71	18.43	18.55	18.56	0.14	100
10^3^	21.86	21.63	21.72	21.74	0.12	100
10^2^	25.02	24.77	24.91	24.90	0.13	100
10^1^	28.21	27.89	28.12	28.07	0.17	100
10^0^	31.12	31.48	31.31	31.30	0.18	100
10^-1^	32.04	33.02	NA	32.53	0.69	67
10^-2^	NA	NA	NA	NA	NA	0
10^-3^	NA	NA	NA	NA	NA	0
NTC	NA	NA	NA	NA	NA	0
**b. 20-replicate testing for the determination of limit of detection.**						
**Concentration** **(copies/ mL)**	**% Detection**	**Mean Ct**	**SD**			
10^1^	100	28.070	0.342			
10^0^	100	31.345	0.455			

**Fig 2:**
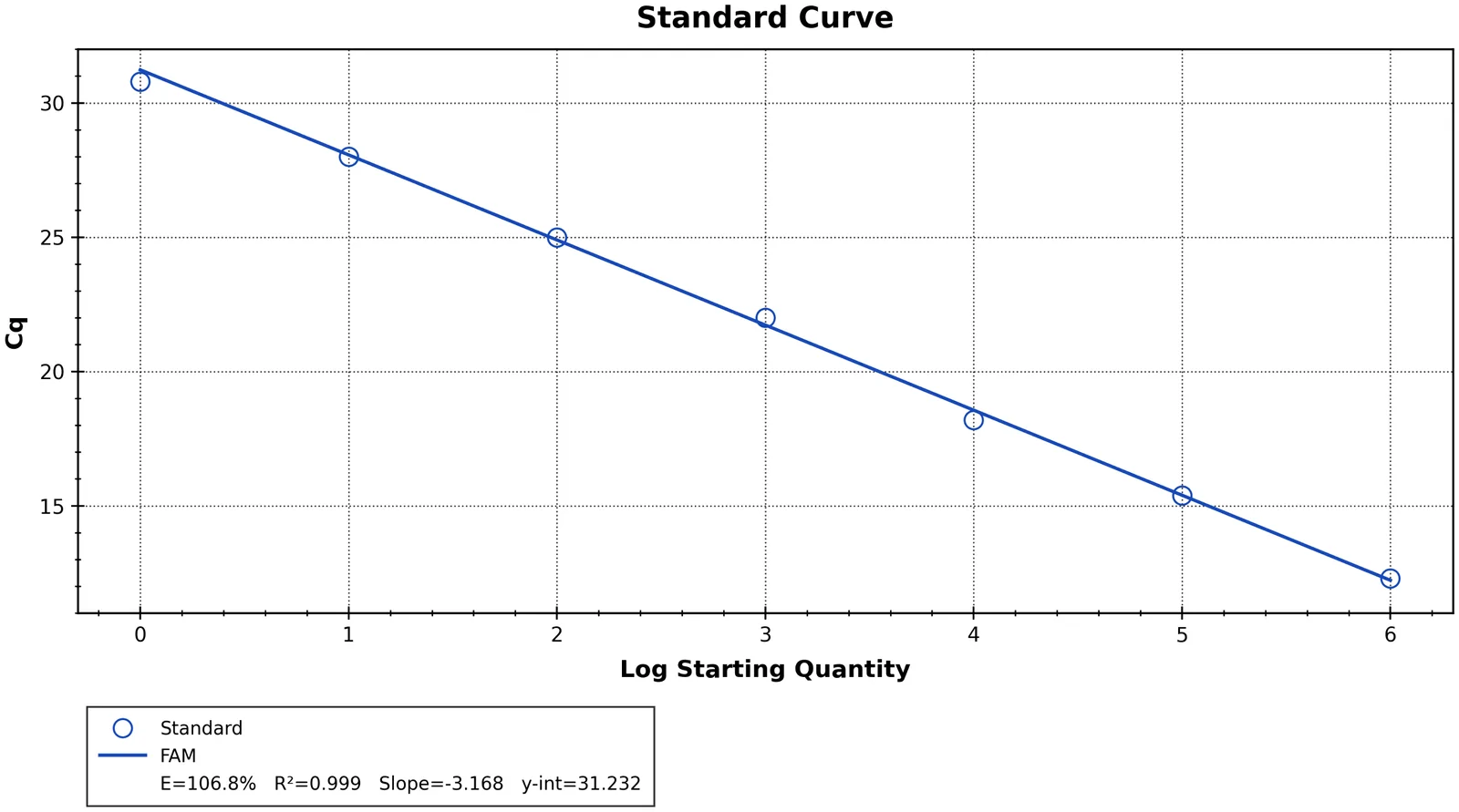
Standard curve and efficiency of the rabies N-gene RT-qPCR assay.

Assay reproducibility was evaluated by performing the optimized protocol within one week of optimization by different personnel using the same reagents and protocol across different real-time PCR platforms. The assay produced consistent results across operators and instruments, with no observable discrepancies.

The optimized RT-qPCR assay was validated against dFAT as the reference method. Validation was performed by extracting RNA from brain and non-brain samples (nasal planum and follicle sinus complexes) collected from dFAT-confirmed positive specimens submitted by RADDLs in Regions 2, 3, 4A, 5, 6, 7, and 11, and the Cordillera Administrative Region (CAR), and testing for rabies viral RNA using the optimized assay. The rabies virus N gene was detected in all corresponding brain and non-brain samples from dFAT-positive cases (Table 9).

**Table 9:** Detection of rabies N gene in 20 DFAT-positive brain and its corresponding nasal planum and follicle sinus complexes.

Sample ID	Ct value		
	Brain	Follicle sinus complex	Nasal planum
RADDL3–22-L097	12.70	28.68	27.22
RADDL5-21-136	14.33	24.57	22.52
RADDL7-21-149	15.48	24.69	26.55
RADDL2-22-9074	16.12	26.25	23.27
RADDL5-21-140	16.70	26.31	27.22
RADDL6-21-4342	17.33	27.96	27.17
RADDL2-22-9034	17.45	26.46	26.58
RADDL11-22-001	18.82	25.91	18.20
CAR-22–020	18.83	24.88	24.08
CAR-22–018	19.28	25.75	22.70
RADDL11-22-002	19.72	26.26	22.19
RADDL4A-22–203	19.90	22.37	18.45
RADDL3-22-0112	20.40	26.23	26.11
RADDL5-22-008	20.95	26.06	27.14
RADDL6-21-4375	21.34	27.09	25.69
RADDL4A-22–171	21.88	25.06	25.81
RADDL7-21-161	22.03	27.86	25.23
RADDL6-21-4343	22.10	24.94	26.18
RADDL2-22-5813	22.67	28.10	30.10
RADDL3-22-0219	27.04	29.14	26.80

Comparison of Ct values among paired brain, follicle sinus complex, and nasal planum specimens using the Friedman test revealed a statistically significant difference among specimen types (p = 7.14 × 10 ⁻ ^6^), indicating that specimen type significantly influenced assay detection performance.

Furthermore, among the dFAT-negative brain samples, 27 of 30 showed concordant negative results by RT-qPCR (Table 10). As shown in Table 11, the assay demonstrated a positive percent agreement (PPA) of 100% (20/20; 95% CI: 83.2–100%), a negative percent agreement (NPA) of 90.0% (27/30; 95% CI: 73.5–97.9%).

**Table 10:** Detection of rabies N gene in DFAT negative brain samples.

Sample ID	qPCR result	Ct value	n
Z-22-014, Z-22-015, Z-22-018, Z-22-042, Z-22-043, Z-22-045, Z-22-051, Z-22-054, Z-22-055, Z-22-057, Z-22-059, Z-22-063, Z-22-064, Z-22-071, Z-22-076, Z-22-077, Z-22-079, Z-22-080, Z-22-093, Z-22-095, Z-22-097, Z-22-098, Z-22-099, Z-22-106, Z-22-109, Z-22-114, Z-22-117	Negative	NA	27
Z-22-022; Z-22-049; Z-22-081	Positive	30.46; 27.91; 32.95	3

**Table 11:** Diagnostic agreement of the RT-qPCR assay compared with the direct fluorescent antibody test (dFAT).

Metric	Concordant / Total	% Agreement	95% CI
Positive Percent Agreement (PPA)	20 / 20	100	83.2–100%
Negative Percent Agreement (NPA)	27 / 30	90.0	73.5–97.9%

*Exact (Clopper–Pearson) binomial confidence intervals were calculated for all percent agreement estimates.

Despite the significant differences in Ct values among specimen types, Spearman's rank correlation analysis (Table 12) demonstrated no significant correlation between brain Ct values and those obtained from follicle sinus complex (ρ = 0.197, p = 0.405) or nasal planum specimens (ρ = 0.074, p = 0.757). These findings indicate that while RT-qPCR consistently detected rabies virus RNA across specimen types, quantitative Ct values in non-brain tissues did not correlate with those observed in paired brain specimens. This is further demonstrated by amplification curves of paired specimens in Figure 3.

**Table 12:** Spearman's rank correlation between RT-qPCR Ct values of brain tissue and non-brain specimens.

Comparison	n	Spearman's ρ	*p*-value	Interpretation
Brain vs. Follicle sinus complex	20	0.197	0.405	No significant correlation
Brain vs. Nasal planum	20	0.074	0.757	No significant correlation

**Fig 3:**
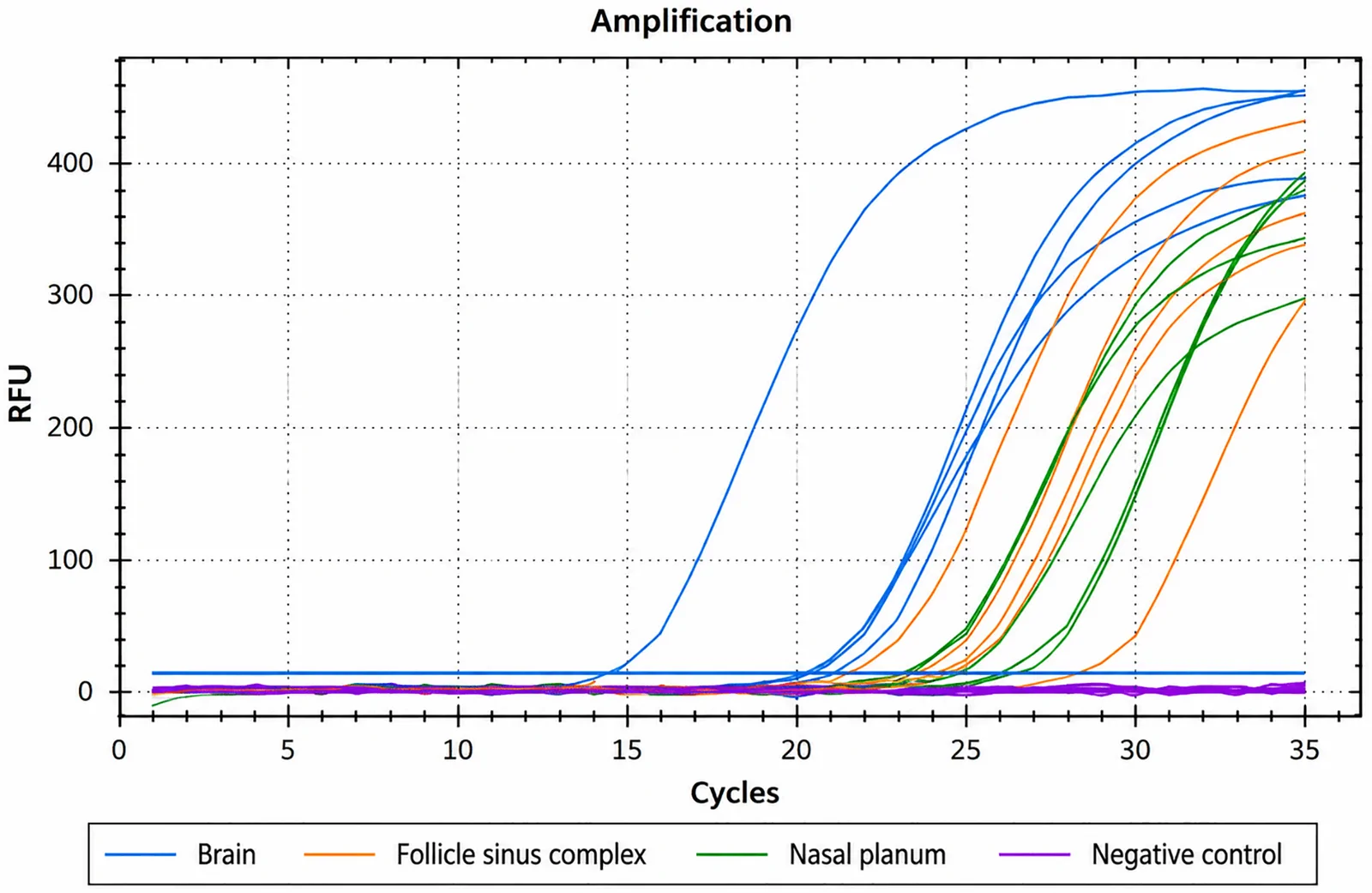
Representative amplification curve for brain, follicle sinus complex, and nasal planum from 5 samples.

## Discussion

The persistence of human rabies cases in the Philippines highlights ongoing limitations in animal rabies surveillance and diagnostic confirmation. Although laboratory diagnosis in animals is critical for guiding post-exposure prophylaxis and monitoring transmission, antemortem specimen collection from suspected rabid animals is often impractical. As the disease progresses, affected animals commonly develop severe agitation and aggression, complicating safe handling and restraint and increasing the risk of occupational exposure to infectious saliva through bites or scratches [27]. These biosafety and logistical constraints necessitate continued reliance on post-mortem diagnosis, supporting the application of the locally tailored RT-qPCR assay evaluated in this study for routine surveillance in resource-limited settings.

Consistent with current recommendations from the World Organization for Animal Health (WOAH), which endorse RT-PCR–based assays for routine rabies diagnostics [28], this study developed an RT-qPCR assay targeting the rabies virus nucleoprotein (N) gene. Selection of the N gene aligns with its high sequence conservation and established utility for both diagnostic detection and molecular epidemiology [19,22,29,30]. Importantly, the assay was built upon primers and probes derived from a Philippine rabies virus isolate (SEA4 clade) [19–22], addressing concerns that genetic variability among rabies virus lineages may compromise the performance of broadly designed molecular assays when applied in endemic settings [19,20]. By optimizing detection for locally circulating strains, the assay directly supports the study objective of improving diagnostic sensitivity and surveillance accuracy within the Philippine rabies context.

Assay optimization was guided by an already established protocol [25] and predefined performance criteria to ensure methodological rigor and consistency with intended diagnostic use. An annealing temperature of 60 °C was identified as optimal based on the lowest mean Ct values (efficient amplification), 100% detection (maintained sensitivity), minimal inter-replicate variability (best reproducibility), and absence of non-specific amplification (high specificity). Although lower annealing temperatures produced comparable Ct values, they resulted in late non-specific signals in non-rabies samples, consistent with known effects of reduced primer specificity under suboptimal thermal conditions [25,31]. Limiting amplification to 35 cycles further enhanced assay specificity by minimizing primer dimer formation and background signal amplification [32,33]. Together, these optimization steps established a robust assay suitable for routine diagnostic workflows.

The optimized RT-qPCR assay demonstrated high analytical sensitivity, with an amplification efficiency of 106.8% and excellent linearity across a broad dynamic range, meeting accepted performance benchmarks for quantitative PCR assays [34]. The determined limit of detection of 1 RNA copy/µL at a 95% confidence level underscores the assay’s ability to detect low-level viral RNA. This level of sensitivity is particularly relevant in the Philippine setting, where delays in sample transport, high ambient temperatures, and variable cold-chain conditions frequently compromise specimen integrity and reduce the sensitivity of antigen-based assays such as dFAT [3,11,12].

A central objective of this study was to evaluate the diagnostic utility of non-brain specimens as alternatives when brain tissue is degraded, insufficient, or unavailable. Validation results demonstrated complete concordance among RT-qPCR results from brain, FSC, and NP specimens and dFAT results from brain specimens of rabies-positive dog (PPA = 100%), confirming the assay’s capability to detect rabies virus RNA across specimen types. However, quantitative differences in Ct values among paired specimens were observed. Statistical comparison using the Friedman test revealed a significant difference in Ct values among brain, follicle sinus complex, and nasal planum samples (p = 7.14 × 10 ⁻ ⁶), indicating that specimen type significantly influenced assay detection performance; indicating that these non-brain specimens are good alternatives, and not replacements for the brain tissue. Additionally, no significant correlation was identified between Ct values obtained from brain tissue and those from either non-brain specimen type. This finding suggests that viral RNA concentrations in peripheral tissues do not directly reflect those present in the central nervous system. Such variability is consistent with the pathogenesis of rabies, in which virus disseminates centrifugally from the central nervous system to peripheral tissues through neural pathways, resulting in heterogeneous viral distribution and replication among anatomical sites [2,35–37].. Moreover, specimen collection was performed under routine field conditions without standardized post-mortem intervals. Variations in the time between death and tissue collection, transport conditions, and RNA preservation may have differentially affected viral RNA integrity across specimen types, potentially contributing to the observed lack of correlation. Nevertheless, the complete positive agreement observed across paired specimens demonstrates that follicle sinus complexes and nasal planum remain reliable qualitative alternative specimens for post-mortem rabies diagnosis when brain tissue is unavailable. Moreover, the consistent detection supports the practical use of nasal planum and follicle sinus complexes for post-mortem diagnosis, particularly when biosafety concerns or logistical constraints limit brain sampling [13–16].

The detection of rabies viral RNA in a small number of dFAT-negative brain samples further underscores the complementary role of molecular diagnostics. A negative percent agreement of 90.0% was observed, reflecting discordance in 3 of 30 dFAT-negative samples. These discordant specimens exhibited high Ct values, indicative of low viral RNA concentrations that may fall below the detection threshold of antigen-based assays. Such discrepancies have been well documented and may arise from uneven viral distribution, early or late stages of infection, or antigen degradation in compromised specimens [38,39]. Nevertheless, because sequencing or an independent molecular confirmatory assay was not performed on these discordant samples, the possibility of false-positive RT-qPCR results cannot be completely excluded. Collectively, these findings reinforce the value of RT-qPCR as a sensitive adjunct to dFAT, particularly in surveillance settings where false-negative results may have important public health consequences.

From a broader surveillance and public health perspective, the assay developed in this study directly supports the global *Zero by 30* initiative, a strategic plan led by the World Health Organization (WHO), World Organization for Animal Health (WOAH), Food and Agriculture Organization (FAO), and the Global Alliance for Rabies Control (GARC) that aims to achieve zero human deaths from dog-mediated rabies by 2030 through strengthened surveillance, improved diagnostic capacity, mass dog vaccination, and timely access to post-exposure prophylaxis [40]. The assay’s high-throughput capability, analytical sensitivity, and compatibility with non-brain specimens position it as a practical tool for enhancing rabies surveillance in the Philippines. Its application may reduce diagnostic delays, improve case confirmation in geographically dispersed regions, and support more accurate monitoring of rabies control efforts.

Several considerations and limitations encountered may inform future work. PCR assay validation was limited to post-mortem dog brain, nasal planum, and follicle sinus complex specimens, and extension to non-canine, and antemortem animal samples may further support surveillance applications. Despite the assay's analytical LoD of 1 RNA copy/µL, further evaluation using a larger panel of non-brain clinical specimens is warranted to establish clinical sensitivity at low viral loads. The lack of correlation between brain and non-brain Ct values also highlights the need to assess low-positive specimens independently. Future studies should characterize assay performance near the detection limit to better define the reliability of nasal planum and follicle-sinus complex specimens. Evaluation of human specimens, such as antemortem saliva and nuchal skin biopsies, may also be valuable, provided appropriate biosafety measures are in place. Specimen collection times following death were not standardized, which may have influenced sample quality; incorporating defined post-mortem intervals in future studies could determine the length of specimen viability and strengthen interpretation under field conditions. Additionally, the absence of an internal extraction and amplification control limits the assessment of sample-specific extraction efficiency and potential amplification inhibition. Although standardized RNA extraction and external controls were employed, these measures cannot exclude such issues in individual specimens. Incorporating an appropriate internal control in future assay development would strengthen quality assurance, subject to optimization and verification to ensure reliable detection of low concentrations of rabies viral RNA. Moreover, larger sample sizes would help to better characterize assay performance across diverse fields and laboratory settings. Finally, adaptation of the probe-based assay to more economical platforms, such as SYBR Green–based formats, could facilitate wider uptake in routine surveillance.

## Conclusion

In conclusion, the RT-qPCR assay developed in this study provides a practical and sensitive diagnostic tool for strengthening rabies surveillance in the Philippines and other endemic settings. By targeting the nucleoprotein gene using primers and probes specifically designed from a Philippine rabies virus isolate, the assay was optimized for the detection of locally circulating rabies virus strains, thereby enhancing its relevance for routine national diagnostics and surveillance. Its ability to reliably detect rabies virus RNA from both brain and selected non-brain specimens supports case confirmation when conventional testing is limited by specimen quality, the need to avoid cranial sampling, or logistical constraints. Although viral RNA concentrations differed among specimen types, the complete positive agreement observed across brain, follicle sinus complex, and nasal planum specimens supports the use of these non-brain tissues as reliable qualitative alternatives for post-mortem diagnosis when brain tissue is unavailable. By expanding specimen options and enabling high-throughput molecular testing, the assay has the potential to improve the timeliness and completeness of animal rabies surveillance, support evidence-based post-exposure prophylaxis decisions, and strengthen monitoring of rabies control programs. Adoption of this assay into routine diagnostic workflows, with further evaluation in human clinical specimens, could enhance the Philippines’ diagnostic capacity and contribute to the country's commitment to the global “*Zero by 30”* initiative to eliminate human deaths from dog-mediated rabies by 2030.
